# Supplementary material for: Modulation of Oncogenic KRAS Signaling by Branched Actin-driven Cell Membrane Protrusions
Source: bioRxiv. 2026 Apr 13:2026.04.09.717047. Preprint. [Version 1] doi: 10.64898/2026.04.09.717047 (PMC13104973; doi:10.64898/2026.04.09.717047)
Supplement: Supplement 1 [file media-1.pdf]

Supplementary Table 1

Plasmid List

Plasmid	Source
pLVX_IRES_Neomycin empty vector	Wesley Burford (UTSW)
pLVX_IRES_Puromycin empty vector	Wesley Burford (UTSW)
pTRE3G empty vector	This study
pLVX:mRuby2-tractin	Wesley Burford (UTSW)
pLVX:CyOFP1-tractin	Wesley Burford (UTSW)
pLVX:mEmerald-tractin	Wesley Burford (UTSW)
pLVX:Arp3-EGFP	Tadamoto Isogai (UTSW)
pLVX:EGFP-KRASG12V	This study
pLVX:SNAP-tag-KRASG12V	This study
pLVX:nSNAP-KRASG12V	This study
pLVX:RBD-cSNAP	This study
pLL7.0: mTiam1(64-437)-tgRFPt-SSPB R73Q	Addgene #60418
pLL7.0: Venus-iLID-CAAX	Addgene #60411
pLVX:EGFP-CAAX	This study
pTRE3G:myr-TIAM1DH/PH	This study
pTRE3G:NF2WT	This study
pTRE3G:NF2S518A	This study
pMD.2g	Addgene #12259
psPax2	Addgene #12260
pSpCas9(BB)-2A-Puro	Addgene # 62988
pMA-Tia1L	Tilmann Bürckstümmer (Horizon Genomics)

nSNAP: Amino terminus (N-terminus) fragment of the SNAP-tag protein (amino acids 1-91)

cSNAP: Carboxyl terminus (C-terminus) fragment of the SNAP-tag protein (amino acids 92-182).

RBD: RAS Binding Domain of the c-RAF1 effector kinase (amin acids 51-131).
