# Supplementary material for: Modulation of Oncogenic KRAS Signaling by Branched Actin-driven Cell Membrane Protrusions
Source: bioRxiv. 2026 Apr 13:2026.04.09.717047. Preprint. [Version 1] doi: 10.64898/2026.04.09.717047 (PMC13104973; doi:10.64898/2026.04.09.717047)
Supplement: Supplement 2 [file media-2.pdf]

Extended Data File 1

ENDOGENOUS KRAS SEQUENCE VERIFICATION

COR-L23 Cell Line

Seq_1: sequence of the Reference WT KRAS DNA (NCBI reference: NM_004985.5)
Seq_2: sequence of the product of a polymerase chain reaction (PCR) targeted at the KRAS endogenous locus

```
Seq_1  1      aagcgtcgatggaggagtttgtaaatgaagtacagttcattacgatacacgtctgcagtc  60
                           |||||||||||||||||||||||||||||||||||||||||||||||
Seq_2  1      -------------ggagtttgtaaatgaagtacagttcattacgatacacgtctgcagtc  47


Seq_1  61     aactggaattttcatgattgaattttgtaaggtattttgaaataatttttcatataaagg  120
              ||||||||||||||||||||||||||||||||||||||||||||||||||||||||||||
Seq_2  48     aactggaattttcatgattgaattttgtaaggtattttgaaataatttttcatataaagg  107


Seq_1  121    tgagtttgtattaaaaggtactggtggagtatttgatagtgtattaaccttatgtgtgac  180
              ||||||||||||||||||||||||||||||||||||||||||||||||||||||||||||
Seq_2  108    tgagtttgtattaaaaggtactggtggagtatttgatagtgtattaaccttatgtgtgac  167

                                                                M  T  E
Seq_1  181    atgttctaatatagtcacattttcattatttttattataagGCCTGCTGAAAATGACTGA  240
              ||||||||||||||||||||||||||||||||||||||||||||||||||||||||||||
Seq_2  168    atgttctaatatagtcacattttcattatttttattataaggcctgctgaaaATGACTGA  227
                                                                M  T  E

               Y  K  L  V  V  V  G  A  G  G  V  G  K  S  A  L  T  I  Q  L
Seq_1  241    ATATAAACTTGTGGTAGTTGGAGCTGGTGGCGTAGGCAAGAGTGCCTTGACGATACAGCT  300
              |||||||||||||||||||||||||| |||||||||||||||||||||||||||||||||
Seq_2  228    ATATAAACTTGTGGTAGTTGGAGCTGTTGGCGTAGGCAAGAGTGCCTTGACGATACAGCT  287
               Y  K  L  V  V  V  G  A  V  G  V  G  K  S  A  L  T  I  Q  L

               I  Q  N  H  F  V  D  E  Y  D  P  T  I  E
Seq_1  301    AATTCAGAATCATTTTGTGGACGAATATGATCCAACAATAGAGgtaaatcttgttttaat  360
              ||||||||||||||||||||||||||||||||||||||||||||||||||||||||||||
Seq_2  288    AATTCAGAATCATTTTGTGGACGAATATGATCCAACAATAGAGgtaaatcttgttttaat  347
               I  Q  N  H  F  V  D  E  Y  D  P  T  I  E

Seq_1  361    atgcatattactggtgcaggaccattctttgatacagataaaggtttctctgaccatttt  420
              ||||||||||||||||||||||||||||||||||||||||||||||||||||||||||||
Seq_2  348    atgcatattactggtgcaggaccattctttgatacagataaaggtttctctgaccatttt  407


Seq_1  421    catgagtacttattacaagataattatgctgaaagttaagttatctgaaatgtaccttgg  480
              |||
Seq_2  408    cat---------------------------------------------------------  410
```

Extended Data File 1

SU.86.86 Cell Line

Seq_1: sequence of the Reference WT KRAS DNA (NCBI reference: NM_004985.5)

Seq_2: sequence of the product of a polymerase chain reaction (PCR) targeted at the KRAS endogenous locus

```
Seq_1  1      aagcgtcgatggaggagtttgtaaatgaagtacagttcattacgatacacgtctgcagtc  60
                           |||||||||||||||||||||||||||||||||||||||||||||||
Seq_2  1      -------------ggagtttgtaaatgaagtacagttcattacgatacacgtctgcagtc  47


Seq_1  61     aactggaattttcatgattgaattttgtaaggtattttgaaataatttttcatataaagg  120
              ||||||||||||||||||||||||||||||||||||||||||||||||||||||||||||
Seq_2  48     aactggaattttcatgattgaattttgtaaggtattttgaaataatttttcatataaagg  107


Seq_1  121    tgagtttgtattaaaaggtactggtggagtatttgatagtgtattaaccttatgtgtgac  180
              ||||||||||||||||||||||||||||||||||||||||||||||||||||||||||||
Seq_2  108    tgagtttgtattaaaaggtactggtggagtatttgatagtgtattaaccttatgtgtgac  167

                                                                   M  T  E
Seq_1  181    atgttctaatatagtcacattttcattatttttattataagGCCTGCTGAAAATGACTGA  240
              ||||||||||||||||||||||||||||||||||||||||||||||||||||||||||||
Seq_2  168    atgttctaatatagtcacattttcattatttttattataagGCCTGCTGAAAATGACTGA  227
                                                                   M  T  E

                Y  K  L  V  V  V  G  A  G  G  V  G  K  S  A  L  T  I  Q  L
Seq_1  241    ATATAAACTTGTGGTAGTTGGAGCTGGTGGCGTAGGCAAGAGTGCCTTGACGATACAGCT  300
              |||||||||||||||||||||||||| |||||||||||||||||||||||||||||||||
Seq_2  228    ATATAAACTTGTGGTAGTTGGAGCTGATGGCGTAGGCAAGAGTGCCTTGACGATACAGCT  287
                Y  K  L  V  V  V  G  A  D  G  V  G  K  S  A  L  T  I  Q  L

                I  Q  N  H  F  V  D  E  Y  D  P  T  I  E
Seq_1  301    AATTCAGAATCATTTTGTGGACGAATATGATCCAACAATAGAGgtaaatcttgttttaat  360
              ||||||||||||||||||||||||||||||||||||||||||||||||||||||||||||
Seq_2  288    AATTCAGAATCATTTTGTGGACGAATATGATCCAACAATAGAGgtaaatcttgttttaat  347
                I  Q  N  H  F  V  D  E  Y  D  P  T  I  E

Seq_1  361    atgcatattactggtgcaggaccattctttgatacagataaaggtttctctgaccatttt  420
              ||||||||||||||||||||||||||||||||||||||||||||||||||||||||||||
Seq_2  348    atgcatattactggtgcaggaccattctttgatacagataaaggtttctctgaccatttt  407


Seq_1  421    catgagtacttattacaagataattatgctgaaagttaagttatctgaaatgtaccttgg  480
              |||||||||
Seq_2  408    catgagtac---------------------------------------------------  416
```

Extended Data File 1

MIA PaCa-2 Cell Line
Seq_1: sequence of the Reference WT KRAS DNA (NCBI reference: NM_004985.5)
Seq_2: sequence of the product of a polymerase chain reaction (PCR) targeted at the KRAS endogenous locus

```
Seq_1  1      aagcgtcgatggaggagtttgtaaatgaagtacagttcattacgatacacgtctgcagtc  60
                          ||||||||||||||||||||||||||||||||||||||||||||||||
Seq_2  378    ------------aggagtttgtaaatgaagtacagttcattacgatacacgtctgcagtc  331


Seq_1  61     aactggaattttcatgattgaattttgtaaggtattttgaaataatttttcatataaagg  120
              ||||||||||||||||||||||||||||||||||||||||||||||||||||||||||||
Seq_2  330    aactggaattttcatgattgaattttgtaaggtattttgaaataatttttcatataaagg  271


Seq_1  121    tgagtttgtattaaaaggtactggtggagtatttgatagtgtattaaccttatgtgtgac  180
              ||||||||||||||||||||||||||||||||||||||||||||||||||||||||||||
Seq_2  270    tgagtttgtattaaaaggtactggtggagtatttgatagtgtattaaccttatgtgtgac  211


                                                                  M  T  E
Seq_1  181    atgttctaatatagtcacattttcattatttttattataagGCCTGCTGAAAATGACTGA  240
              ||||||||||||||||||||||||||||||||||||||||||||||||||||||||||||
Seq_2  210    atgttctaatatagtcacattttcattatttttattataaggcctgctgaaaATGACTGA  151
                                                                  M  T  E


               Y  K  L  V  V  V  G  A  G  G  V  G  K  S  A  L  T  I  Q  L
Seq_1  241    ATATAAACTTGTGGTAGTTGGAGCTGGTGGCGTAGGCAAGAGTGCCTTGACGATACAGCT  300
              ||||||||||||||||||||||||| ||||||||||||||||||||||||||||||||||
Seq_2  150    ATATAAACTTGTGGTAGTTGGAGCTTGTGGCGTAGGCAAGAGTGCCTTGACGATACAGCT  91
               Y  K  L  V  V  V  G  A  C  G  V  G  K  S  A  L  T  I  Q  L


               I  Q  N  H  F  V  D  E  Y  D  P  T  I  E
Seq_1  301    AATTCAGAATCATTTTGTGGACGAATATGATCCAACAATAGAGgtaaatcttgttttaat  360
              ||||||||||||||||||||||||||||||||||||||||||||||||||||||||||||
Seq_2  90     AATTCAGAATCATTTTGTGGACGAATATGATCCAACAATAGAGgtaaatcttgttttaat  31
               I  Q  N  H  F  V  D  E  Y  D  P  T  I  E


Seq_1  361    atgcatattactggtgcaggaccattctttgatacagataaaggtttctctgaccatttt  420
              ||||||||||||||||||||||||||||||
Seq_2  30     atgcatattactggtgcaggaccattctttt------------------------------  1


Seq_1  421    catgagtacttattacaagataattatgctgaaagttaagttatctgaaatgtaccttgg  480

Seq_2  0      ------------------------------------------------------------  1
```

Extended Data File 1: Verification of KRAS mutation at codon 12 in patient-derived cell lines. The PCR product sequence was aligned to a refence wildtype KRAS4B sequence (NCBI reference: NM_004985.5) to validate the KRAS mutations at codon 12. For every sequence, the first methionine codon is highlighted in green. Codon 12 is highlighted in blue and magenta for the reference and PCR sequences, respectively. Yellow highlights the amino acid at codon 12.
